# Compound K, a Metabolite of Ginseng Saponin, Induces Mitochondria-Dependent and Caspase-Dependent Apoptosis via the Generation of Reactive Oxygen Species in Human Colon Cancer Cells

**DOI:** 10.3390/ijms11124916

**Published:** 2010-12-01

**Authors:** In Kyung Lee, Kyoung Ah Kang, Chae Moon Lim, Ki Cheon Kim, Hee Sun Kim, Dong Hyun Kim, Bum Joon Kim, Weon Young Chang, Jae Hyuck Choi, Jin Won Hyun

**Affiliations:** 1 Department of Microbiology and Cancer Research Institute, Seoul National University College of Medicine, Seoul 110-799, Korea; E-Mails: inkyeong@korea.ac.kr (I.K.L.); kbumjoon@snu.ac.kr (B.J.K.); 2 School of Medicine, Jeju National University, Jeju 690-756, Korea; E-Mails: legna48@hanmail.net (K.A.K.); eric2550@hanmail.net (C.M.L.); svv771@hotmail.com (K.C.K.); orkorea@jejunu.ac.kr (W.Y.C.); 3 Department of Neuroscience, College of Medicine, Ewha Womans University, Seoul 110-783, Korea; E-Mail: hskim@ewha.ac.kr; 4 Department of Microbial Chemistry, College of Pharmacy, Kyung Hee University, Seoul 130-701, Korea; E-Mail: dhkim@khu.ac.kr

**Keywords:** Compound K, reactive oxygen species, mitochondrial membrane potential, c-Jun NH_2_-terminal kinase, p38 mitogen-activated protein kinase

## Abstract

The objective of this study was to elucidate the cytotoxic mechanism of Compound K, with respect to the involvement of reactive oxygen species (ROS) and the mitochondrial involved apoptosis, in HT-29 human colon cancer cells. Compound K exhibited a concentration of 50% growth inhibition (IC_50_) at 20 μg/mL and cytotoxicity in a time dependent manner. Compound K produced intracellular ROS in a time dependent fashion; however, *N*-acetylcysteine (NAC) pretreatment resulted in the inhibition of this effect and the recovery of cell viability. Compound K induced a mitochondria-dependent apoptotic pathway via the modulation of Bax and Bcl-2 expressions, resulting in the disruption of the mitochondrial membrane potential (Δψ_m_). Loss of the Δψ_m_ was followed by cytochrome c release from the mitochondria, resulting in the activation of caspase-9, -3, and concomitant poly ADP-ribosyl polymerase (PARP) cleavage, which are the indicators of caspase-dependent apoptosis. The apoptotic effect of Compound K, exerted via the activation of c-Jun NH_2_-terminal kinase (JNK) and p38 mitogen-activated protein kinase (MAPK), was abrogated by specific MAPK inhibitors. This study demonstrated that Compound K-mediated generation of ROS led to apoptosis through the modulation of a mitochondria-dependent apoptotic pathway and MAPK pathway.

## 1. Introduction

**Figure 1.**
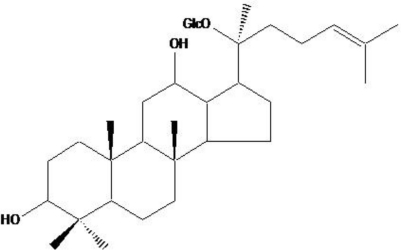
Chemical structure of Compound K, [20-*O*-d-glucopyranosyl-20(*S*)-protopanaxadiol].

Reactive oxygen species (ROS) are the by-products of normal cellular oxidative processes, and are mainly generated in the mitochondria. They attack lipid membranes, proteins, and DNA, leading to serious cell damage, and regulate apoptotic signal transduction [1–3]. Indeed, ROS induce the depolarization of the mitochondrial membrane, and lead to increased levels of pro-apoptotic molecules in the cytosol [4–6]. Apoptosis is followed by cell shrinkage, nuclear fragmentation, membrane blebbing, DNA fragmentation, and finally the breakdown of the cell into apoptotic bodies [7–9]. Capases, a family of cysteine-dependent aspartate-directed proteases, play a critical role in the initiation and execution of apoptosis [10–12]. Among this family, caspase-9 and -3 are the most crucial for the initiation and execution of apoptosis in various cell types [13,14]. Cancer is a disease that involves excessive proliferation of cells and insufficient cell suicide via apoptotic process. [20-*O*-(β-d-glucopyranosyl)-20(*S*)-protopanaxadiol] (Compound K, Figure 1) is the main metabolite of protopanaxadiol-type ginsenoside formed in the intestine after oral administration [15–18]. We recently reported that Compound K exhibited cytotoxicity through the induction of apoptosis, arrest at the G_1_ phase of cell cycle, and inhibition of telomerase activity in human leukemia cells [19–21]; that the combined treatment of Compound K and radiation enhanced the cell death in human lung cancer cells [22]; and that Compound K induced apoptosis in MCF-7 breast cancer cells through the modulation of AMP-activated protein kinase [23]. The gastrointestinal tract, especially the colon, is constantly exposed to ROS originating from endogenous and exogenous sources [24]. Colorectal cancer is the fourth most prevalent carcinoma in western society and the second cause of cancer death [25]. And genetic alterations by ROS are the ultimate underlying mechanisms of colorectal carcinogenesis [26,27]. Compound K has been shown to exhibit anti-proliferative effects on colon cancer cells, which was mediated through apoptosis [28–30]. Despite evidence of its anti-proliferative effects in colon cancer, the cytotoxic mechanism of this effect with respect to the involvement of ROS and mitochondrial involved apoptosis, has not been investigated. Our study showed that Compound K significantly induced ROS generation, which in turn led to apoptotic signals including mitochondria-dependent and caspase-dependent processes.

## 2. Results and Discussion

### 2.1. ROS-Induced Cytotoxic Effect of Compound K on HT-29 Colon Cancer Cells

**Figure 2.**
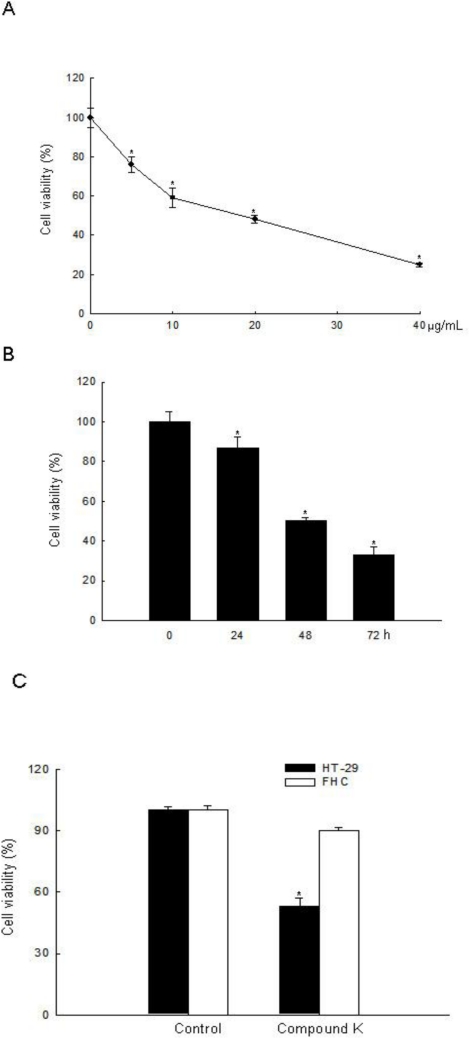
Cytotoxic effect of Compound K in human colon cells. Cell viability (**A**) at the indicated concentrations of Compound K at 48 h in HT-29 cancer cells; (**B**) at the indicated times with Compound K at 20 μg/mL in HT-29 cells; (**C**) at 20 μg/mL of Compound K in FHC normal colon cells and HT-29 colon cancer cells was assessed using MTT test. *Significantly different from control (p < 0.05).

**Figure 3.**
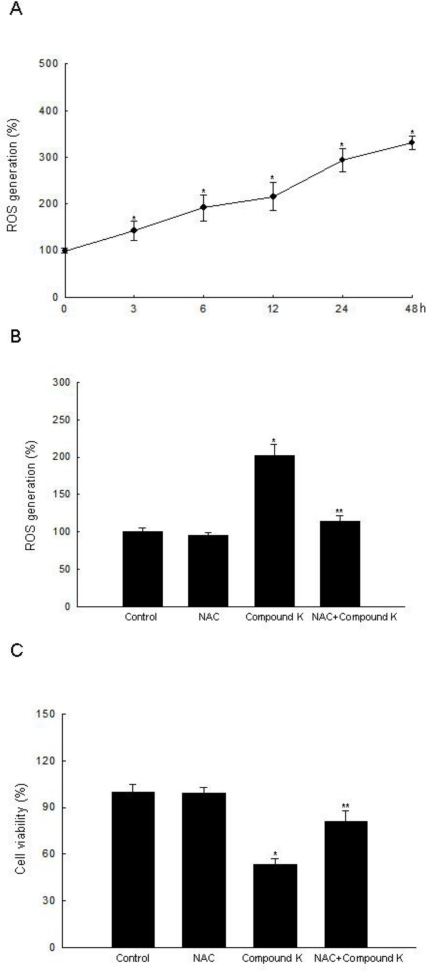
Intracellular ROS generation induced by Compound K treatment. (**A**) Intracellular ROS generated by Compound K were detected at indicated times by a spectrofluorometer after DCF-DA treatment. (**B**) After treatment with NAC and/or Compound K, intracellular ROS were detected at 24 h by spectrofluorometer after DCF-DA treatment. (**C**) After treatment with NAC and/or Compound K, cell viability was assessed by MTT assay. *Significantly different from control (p < 0.05), and **significantly different from Compound K-treated cells (p < 0.05).

Compound K is an active metabolite of ginsenosides and exhibits anti-tumor effects against various types of cancer cells [16,17,19–23,28–36]. In the present study, we investigated the effects and mechanism of action of Compound K in ROS-mediated apoptosis in HT-29 cancer cells. Although it had been previously shown that Compound K induced apoptosis via a Ca^2+^/calmodulinactivated protein kinase-IV/AMP-activated protein kinase pathway in HT-29 colon cancer cells [28,29], Compound K-induced ROS-mediated apoptosis in colon cancer cells had not been investigated. Several anticancer agents used in the treatment of cancer have been shown to cause increased cellular ROS generation [37–39]. Compound K inhibited HT-29 cell growth in a dose-dependent manner at 10, 20, 30, and 40 μg/mL at 48 h, and the concentration effecting 50% growth inhibition (IC_50_) was 20 μg/mL (Figure 2A). Compound K at 20 μg/mL also inhibited HT-29 cell growth in a time-dependent manner (Figure 2B), but did not exhibit cytotoxicity in FHC normal colon cells compared to HT-29 cells at day 2 (Figure 2C). Intracellular ROS, as signaling intermediates, are involved in cell death signal transduction pathways. Compound K induced ROS generation as compared to control in a time-dependent manner (Figure 3A), and NAC, a distinct antioxidant and ROS scavenger, exerted a scavenging effect on the ROS generated by Compound K (Figure 3B). Subsequently, NAC significantly abolished the Compound K-triggered cell death; cell survival was increased to 81% in NAC-pretreated, Compound K-treated cells, compared to 53% in cells treated only with Compound K (Figure 3C). This result suggests that the ROS generated by Compound K induces the cell death.

### 2.2. Induction of Apoptosis by Compound K via a Mitochondria-Dependent Pathway

**Figure 4.**
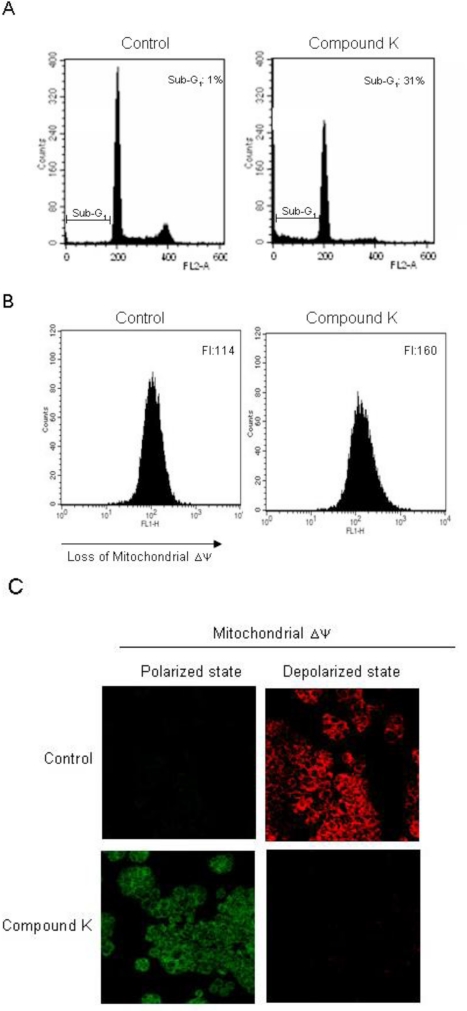

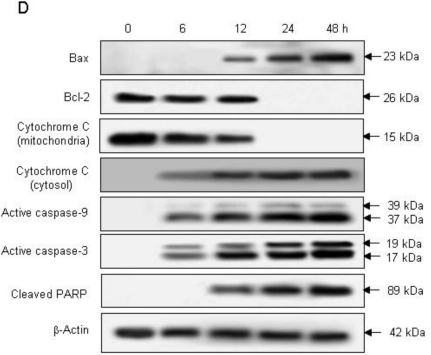
Induction of mitochondria-dependent and caspase-dependent apoptosis by Compound K treatment. (**A**) Apoptotic sub-G_1_ cells were detected by flow cytometry after PI staining. (**B**) Δψ_m_ was analyzed by flow cytometry and (**C**) confocal microscopy after staining cells with JC-1 dye. (**D**) Cell lysates were electrophoresed and Bax, Bcl-2, cytochrome c, active caspase-9, active caspase-3, and cleaved PARP proteins were detected using their corresponding antibodies.

Such increases in intracellular ROS cause loss of mitochondria membrane permeability, resulting in the induction of apoptosis [40,41]. We investigated whether the cytotoxicity of Compound K was associated with the induction of apoptosis. Sub G_1_-hypodiploid cells, which are an indicator of apoptosis, increased in Compound K-treated cells as compared to control cells (Figure 4A). Apoptotic pathway requires the alteration of the mitochondrial membrane potential (Δψ_m_), which leads to mitochondrial membrane permeabilization and is followed by a release of cytochrome c and caspases activation [4,42]. Compound K-treated cells exhibited a loss of Δψ_m_, as substantiated by an increase of fluorescence intensity in fluorescence (FL-1) using the JC-1 dye (Figure 4B). In non-apoptotic cells, the monomer form of JC-1 accumulates as aggregates in the mitochondria, which then emits red fluorescence; whereas in apoptotic cells, JC-1 does not accumulate and remains a monomer, emitting green fluorescence. The control cells exhibited strong red fluorescence in the mitochondria; however, Compound K-treated cells resulted in a decreased red fluorescence in the mitochondria and increased green fluorescence, suggesting that Compound K treatment disrupted the mitochondrial Δψ (Figure 4C). During the apoptotic process, Bcl-2, an anti-apoptotic regulator, prevents the opening of the mitochondrial membrane pores, whereas Bax, an apoptotic regulator, induces it [43]. Compound K was shown to increase Bax expression while decreasing that of Bcl-2 expression. Therefore, Compound K-induced loss of the Δψ_m_ may have been a result of an up-regulation of Bcl-2 and a down-regulation of Bax. The pore opening induces the loss of the Δψ_m_, which in turn induces the release of cytochrome c from the mitochondria [44,45]. Compound K induced the release of cytochrome c from mitochondria into cytosol (Figure 4D). The mitochondrial membrane disruption by Compound K activated caspase-9 (37 and 39 kDa) and caspase-3 (19 and 17 kDa), a target of caspase-9, which was further demonstrated by PARP cleavage (89 kDa) (Figure 4D). These results suggest that Compound K induced apoptosis via a caspase--dependent pathway in the mitochondria. Further, Compound K-induced decreases in the Bcl-2 protein and corresponding increases in the Bax protein, results in the opening of mitochondrial membrane pores, facilitating the release of cytochrome c from mitochondria into cytosol. Cytochrome c is bound to the outer surface of the inner membrane phospholipids. An early process in the release of cytochrome c is its dissociation from the inner membrane. Mitochondrial ROS have been shown to promote cytochrome c release to the cytosol by dissociation from these membrane phospholipids [46,47]. Therefore, Compound K-mediated ROS production may target membrane phospholipids resulting in dissociation of cytochrome c.

### 2.3. Induction of Apoptosis by Compound K via JNK and p38 MAPK Activation

**Figure 5.**
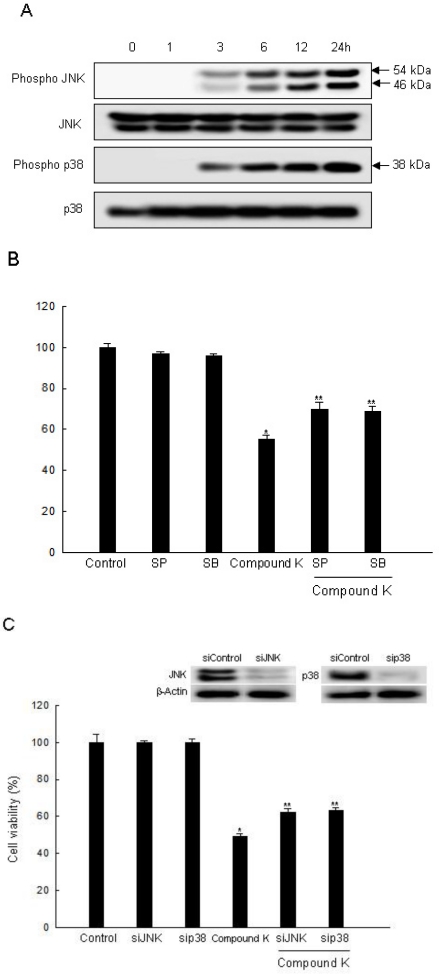
Effect of Compound K on the MAPK signaling pathway. (**A**) Cell lysates were electrophoresed and were immunoblotted using anti-JNK, -phospho JNK, -p38, and -phospho p38 antibodies. (**B**) After treatment with MAPK inhibitors or/and Compound K, cell viability was assessed by MTT assay. (**C**) After transfection of siRNA against MAPK, and/or Compound K, cell viability was assessed by MTT assay. *Significantly different from control (p < 0.05), and **significantly different from Compound K-treated cells (p < 0.05).

Various studies have suggested a possible mechanism for the JNK and p38 MAPK pathway, as related to mitochondrial depolarization and apoptosis induction [48]. The activated MAPK family members including the phosphorylated form of p38, JNK, and ERK are common components of the apoptotic process [49–51]. Some reports have shown that ROS acted as upstream regulators, resulting in the activation of p38 MAPK and JNK [52,53]. Intracellular ROS are also upstream of AMP-activated protein kinase (AMPK) activation [54]. AMPK is highly sensitive to oxidative stress because increased cellular ROS change the AMP level, which leads to rapid AMPK activation. The involvement of AMPK in the inhibition of carcinogenesis can modulate the regulation of COX-2 [55]. Our previous study demonstrated that Compound K exhibited apoptosis by ROS-mediated AMPK activation in MCF-7 breast cancer cells [23]. In the present study, Compound K induced the activation of JNK and p38 MAPK in a time-dependent manner (Figure 5A). We then examined whether a specific inhibitor of JNK and p38 MAPK could attenuate cell death through activation of JNK and p38 MAPK signaling by Compound K. Results revealed that SP600125 (an inhibitor of JNK) and SB203580 (an inhibitor of p38 MAPK) attenuated the cytotoxic effect of Compound K (Figure 5B). Likewise, siJNK and sip38-transfected cells abolished the cytotoxic effect of Compound K (Figure 5C). Our results revealed that phospho p38 MAPK and phospho JNK were notably increased after Compound K treatment. In contrast, Compound K treatment reduced the level of phospho ERK expression (data not shown). This suggested that activation of p38 MAPK and JNK was related to Compound K-induced apoptotic cell death. These results indicate that JNK and p38 MAPK may play a role in Compound K-induced cytotoxicity in HT-29 cells.

## 3. Experimental Section

### 3.1. Chemicals

Compound K was provided by professor Dong Hyun Kim (Kyung Hee University, Seoul, Korea). [3-(4,5-dimethylthiazol-2-yl)-2,5-diphenyltetrazolium] bromide (MTT), 2′,7′-dichlorodihydrofluorescein diacetate (DCF-DA), *N*-acetylcysteine (NAC), propidium iodide, SP600125, and SB203580 were purchased from Sigma Chemical Co. (St. Louis, MO, USA). 5,5′,6,6′-Tetrachloro-1,1′,3,3′-tetraethylbenzimidazolylcarbocyanine chloride (JC-1) was purchased from Molecular Probes (Eugene, OR, USA). The primary anti-Bcl-2, -Bax, -cytochrome c, -caspase-9, -caspase-3, -poly ADP-ribosyl polymerase (PARP), -c-Jun NH_2_-terminal kinase (JNK), -phospho JNK, -p38, and -phospho p38 antibodies were purchased from Cell Signaling Technology (Beverly, MA, USA).

### 3.2. Cells and Cell Culture

Human colon adenocarcinoma (HT-29) and normal colon cells (FHC), from the American type culture collection (Rockville, MD, USA), were maintained at 37 °C in an incubator with a humidified atmosphere of 5% CO_2_ and cultured in RPMI 1640 medium containing 10% heat-inactivated fetal calf serum, streptomycin (100 μg/mL), and penicillin (100 units/mL).

### 3.3. Cell Viability Assay

The effect of Compound K on the viability of the cells was determined by the MTT assay, which is based on the reduction of a tetrazolium salt by mitochondrial succinatedehydrogenase in viable cells [56]. The cells were seeded in a 96 well plate at a density of 1 × 10^5^ cells/mL, treated with Compound K, and after incubating for 48 h, 50 μL of the MTT stock solution (2 mg/mL) was added to each well to attain a total reaction volume of 250 μL. After an incubation of 4 h, the supernatants were aspirated. The formazan crystals in each well were then dissolved in 150 μL dimethylsulfoxide, and the absorbance at 540 nm was read on a scanning multi-well spectrophotometer.

### 3.4. Measurement of Intracellular Reactive Oxygen Species (ROS)

The DCF-DA method was used to detect the levels of intracellular ROS [42]. Cells were seeded onto a 96 well plate at 2 × 10^4^ cells/well. The day after plating, the cells were treated with NAC (2 mM) for 30 min and then treated with Compound K for 24 h. After the addition of 25 mM of the DCF-DA solution for 20 min, the fluorescence of 2′,7′-dichlorofluorescein was measured using a Perkin Elmer LS-5B spectrofluorometer.

### 3.5. Detection of Sub-G_1_ Hypodiploid Cells

The amount of apoptotic sub-G_1_ hypodiploid cells was determined by flow cytometry [57]. Cells were treated with Compound K for 48 h. Harvested cells were then washed twice with phosphate buffered saline (PBS) and fixed in 70% ethanol for 30 min at 4 °C. Subsequently, the cells were incubated in 50 mg/mL propidium iodide solution and 50 μg/mL RNase A in the dark for 30 min at 37 °C. A flow cytometric analysis was performed using a FACS Calibur flow cytometer (Becton Dickinson, Mountain View, CA, USA). The sub-G_1_ hypodiploid cells were assessed based on histograms generated by Cell Quest and Mod-Fit computer programs.

### 3.6. Analysis of Mitochondrial Membrane Potential (Δψ_m_)

Cells were stained with JC-1 (10 μg/mL), and were then analyzed using flow cytometry [58]. In addition, for image analysis, the JC-1-stained cells were mounted with mounting medium (DAKO, Carpinteria, CA, USA). Microscopic images were collected using the Laser Scanning Microscope 5 PASCAL program (Carl Zeiss, Jena, Germany) on a confocal microscope.

### 3.7. Western Blot Analysis

Harvested cells were washed in PBS, lysed in a lysis buffer [120 mM NaCl, 40 mM Tris (pH 8), 0.1% NP 40] and then centrifuged at 13,000 × g for 15 min. Aliquots of the lysates (50 μg of protein) were boiled at 95 °C for 5 min and electrophoresed on SDS–polyacrylamide gels. Gels were transferred onto nitrocellulose membranes for blotting (Bio-Rad, CA, USA), and the membranes were then incubated with primary antibodies. The membranes were further incubated with secondary immunoglobulin G-horseradish peroxidase conjugates, and then underwent enhanced chemiluminescence using a Western blotting detection kit (Amersham, Buckinghamshire, UK). The protein bands were visualized using luminescent image analyzer.

### 3.8. Transient Transfection of Small RNA Interference (siRNA)

Cells were seeded at 1.5 × 10^5^ cells/well in 24 well plate and allowed to reach approximately 50% confluence on the day of transfection. The siRNA construct used were obtained as mismatched siRNA control (siControl, Santa Cruz Biotechnology, Santa Cruz, CA, USA), siRNA against JNK, and p38 (Santa Cruz Biotechnology, Santa Cruz, CA, USA). Cells were transfected with 10–50 nM siRNA using lipofectamineTM 2000 (Invitrogen, Carlsbad, CA, USA) based on the manufacturer’s instruction. At 24 h after transfection, the cells were treated with Compound K for 48 h and examined by either Western blot analysis or MTT assay.

### 3.9. Statistical Analysis

All the measurements were performed in triplicate and all values were represented as the mean standard error (SE). Results were subjected to an analysis of the variance (ANOVA) using the Tukey test for analysis of the differences. Statistical significance was set at p > 0.05.

## 4. Conclusions

This study demonstrated that Compound K-mediated generation of ROS led to apoptosis through activation of p38 MAPK and JNK, which modulate the expression of Bcl-2 and Bax, and then trigger loss of mitochondrial membrane potential, cytochrome c release, and caspase activation.
